# A comparative assessment of the community frontline health workers for their knowledge and practices of malaria diagnosis and treatment in three contiguous districts Mandla, Balaghat, and Dindori of Madhya Pradesh, India

**DOI:** 10.1186/s12936-023-04492-8

**Published:** 2023-02-21

**Authors:** Mrigendra P. Singh, Harsh Rajvanshi, Sekh Nisar, Akansha Singh, Himanshu Jayswar, Srinath Singh, R. K. Mehra, S. K. Shrivastava, Ram Shankar Sahu, Brajesh Patel, Ramji Bhalavi, Kalyan B. Saha, Ravendra K. Sharma, Ashok K. Mishra, Harpreet Kaur, Aparup Das, Praveen K. Bharti, Altaf A. Lal

**Affiliations:** 1 Malaria Elimination Demonstration Project, Mandla, Madhya Pradesh India; 2 Indian Council of Medical Research – National Institute of Research in Tribal Health (ICMR-NIRTH), Jabalpur, Madhya Pradesh India; 3 Directorate General of Health Services, Government of Madhya Pradesh, Bhopal, Madhya Pradesh India; 4 Department of Health Services, Government of Madhya Pradesh, Mandla, Madhya Pradesh India; 5 Department of Health Services, Government of Madhya Pradesh, Dindori, Madhya Pradesh India; 6 Department of Health Services, Government of Madhya Pradesh, Balaghat, Madhya Pradesh India; 7 Indian Council of Medical Research – National Institute of Medical Statistics (ICMR-NIMS), New Delhi, India; 8 Indian Council of Medical Research (ICMR), Ministry of Health and Family Welfare, New Delhi, India; 9 Indian Council of Medical Research – National Institute of Malaria Research (ICMR-NIMR), New Delhi, India; 10 Foundation for Disease Elimination and Control of India (FDEC India), Mumbai, Maharashtra India; 11 Present Address: Asia Pacific Leaders Malaria Alliance (APLMA), Singapore, Singapore; 12 Present Address: Department of Health and Family Welfare, NHM Raigarh, Raigarh, Chattisgarh India; 13 Present Address: Department of Economics, Chaudhary Charan Singh University, Meerut, India

**Keywords:** ASHA needs assessment, Mandla, Balaghat, Dindori, Malaria elimination, Capacity building

## Abstract

**Background:**

Global malaria cases rose by 14 million, and deaths by 69,000, in 2020. In India, a 46% decline has been reported between 2020 and 2019. In 2017, the Malaria Elimination Demonstration Project conducted a needs-assessment of the Accredited Social Health Activists (ASHAs) of Mandla district. This survey revealed the inadequate level of knowledge in malaria diagnosis and treatment. Subsequently, a training programme was launched for enhancing malaria-related knowledge of ASHAs. The present study was conducted in 2021 to evaluate the impact of training on malaria-related knowledge and practices of ASHAs in Mandla. This assessment was also done in two adjoining districts: Balaghat and Dindori.

**Methods:**

A cross-sectional survey using a structured questionnaire was administered to ASHAs to measure their knowledge and practices related to malaria etiology, prevention, diagnosis, and treatment. A comparison of information collected from these three districts was performed using simple descriptive statistics, comparison of means and multivariate logistic regression analysis.

**Results:**

Significant improvement was noted amongst ASHAs of district Mandla between 2017 (baseline) and 2021 (endline) in knowledge related to malaria transmission, preventive measures, adherence to the national drug policy, diagnosis using rapid diagnostic tests, and identification of age group-specific, colour-coded artemisinin combination therapy blister packs (p < 0.05). The multivariate logistic regression analysis revealed that odds of Mandla baseline was 0.39, 0.48, 0.34, and 0.07 times lower for malaria-related knowledge on disease etiology, prevention, diagnosis, and treatment, respectively (p < 0.001). Further, participants in districts Balaghat and Dindori showed significantly lower odds for knowledge (p < 0.001) and treatment practices (p < 0.01) compared to Mandla endline. Education, attended training, having a malaria learner’s guide, and minimum 10 years’ work experience were potential predictors for good treatment practices.

**Conclusion:**

The findings of the study unequivocally establishes significant improvement in overall malaria-related knowledge and practices of ASHAs in Mandla as a result of periodic training and capacity building efforts. The study suggests that learnings from Mandla district could be helpful in improving level of knowledge and practices among frontline health workers.

## Background

Compared to 2019, 14 million more cases of malaria were reported in 2020 globally (241 million *vs* 227 million) and 69,000 more people died from malaria in 2020 (627,000 *vs* 558,000). Of those additional deaths, around two-thirds (47,000) were attributed to disruption in malaria services, including prevention, diagnosis and treatment [1]. The majority of these disruptions were reported in sub-Saharan Africa, with a more than 20% reduction in testing for malaria. Long-lasting insecticidal nets (LLIN) distribution targets for 2020 were not achieved in 28% of countries and the activity spilled over to 2021 [1].

India contributed about 83% of estimated malaria cases in the WHO Southeast Asia region [1]. About 44% of reported malaria cases in India was disproportionately contributed by the ~ 25 tribal-dominated districts that comprise 5% of the country’s population [2]. Amongst 11 High Burden High Impact (HBHI) countries, India reported a decrease in malaria cases (46%) in 2020 compared to 2019 [1]. These 11 countries accounted for 70% of global cases and 71% of global deaths in 2020 [1]. India also reported a decline in testing rates of malaria by up to 20% during the COVID-19 Pandemic in 2020 [3]. As of 2021, the distribution of malaria in India was 62% *Plasmodium falciparum*, 37.2% *Plasmodium vivax*, and 0.8% mixed infections of *P. falciparum* and *P. vivax* [1].

The Malaria Elimination Demonstration Project (MEDP) is a first-of-its-kind, public–private-partnership between the Indian Council of Medical Research (ICMR) through the National Institute of Research in Tribal Health (NIRTH), Government of Madhya Pradesh (GoMP), and the Foundation for Disease Elimination and Control of India (FDEC-India, established by Sun Pharmaceutical Industries Ltd as a not-for-profit entity). The goal of MEDP is to demonstrate successful elimination of malaria from 1,233 villages of Mandla district, so that lessons learnt could be used for eliminating malaria from the rest of Madhya Pradesh and the country. As part of MEDP, all Accredited Social Health Activists (ASHAs) in the project areas were engaged in malaria diagnosis and treatment strategies.

ASHAs, are trained community female health workers who perform public health intervention programmes at community level in India. Amongst an array of roles and responsibilities of multiple health programmes, with maternal and child health activities as the principal focus, ASHAs are also responsible for case detection, diagnostic tests, and providing treatment for malaria in their communities [4].

In 2017, MEDP conducted a needs-assessment survey of ASHAs of Mandla district to determine their knowledge and practices for malaria-specific interventions. The study concluded that ASHAs of Mandla district were inadequately trained in malaria diagnosis and treatment [4]. Only 10–15% ASHAs could recognize *P. vivax* and *P. falciparum* rapid diagnostic tests (RDTs) correctly. Their awareness about correct age group for artemisinin combination therapy (ACT) blister packs was poor. The study also revealed that primaquine doses were not administered as per national drug policy for radical treatment of malaria infection. Lack of diagnostics (RDTs) and anti-malarial drugs was also noted, which was a limiting factor for ASHAs to dispense appropriate services to the community [4].

Following this assessment, the district administration of Mandla invited MEDP to conduct one-on-one training for all ASHAs of the district and continue providing on-the-job training of ASHAs using the project’s Village Malaria Workers and Malaria Field Coordinators. The project also developed and distributed a pocket-sized job-aid in Hindi as a quick reference tool for delivering malaria services to all ASHAs of Mandla district [5]. After 36 months of field operations, the project reported a 91% reduction in indigenous malaria transmission and reported zero malaria cases in nine out of total 46 months of the project operations [6]. MEDP also developed a sensitive and robust surveillance system, which could track the origin of most of the imported cases diagnosed in Mandla.

To assess the impact of periodic training of ASHAs particularly on knowledge and practices related to malaria, the project conducted an endline survey of ASHAs in Mandla and two adjoining districts of Balaghat (high malaria endemicity) and Dindori (low malaria endemicity), using a common protocol.

## Methods

### Study sites

District Mandla is situated 80.54º E longitude and 22.63º N latitude. About 45% of the total geographical area is forest covered. About 58% population belong to the ethnic tribal groups *Gond* and *Baiga*. This is a highly malarious district with seasonal transmission caused by *P. falciparum* and *P. vivax* species. *Anopheles culicifacies* is the main malaria vector in the district [7].

District Balaghat is situated 80.18º E longitude and 21.8º N latitude. It is well known for vast tracts of forest populated by particularly the vulnerable tribal group *Baigas*. It is a highly malarious district and has seasonal malaria transmission with peak in post-monsoon (October–November) season mainly caused by *P. falciparum* [8]. Intermittent streams and their tributaries provide potential breeding for *An. culicifacies* and *Anopheles fluviatilis* [8].

District Dindori is geographically situated between 81.07º E longitude and 22.85º N latitude with 40% area forest cover. About 65% population belong to the particularly vulnerable tribal group *Baiga*. This is a low malarious district and has seasonal malaria transmission with peak in monsoon and post-monsoon seasons (August-November) mostly caused by *P. falciparum*. *Anopheles culicifacies* is the main malaria vector in the district [7].

### Study setting

A cross-sectional survey was conducted during initiation of MEDP (baseline survey) in 2017 [4] and repeated at the end (endline survey) in 2021 in district Mandla. Further in 2021, two bordering and tribal-dominated districts, Balaghat and Dindori, were selected purposively as control districts. Before-after (Mandla baseline *vs* endline) and case–control (Mandla endline *vs* Balaghat and Dindori) design was adapted for comparison of the results from these three districts.

A fully structured, pre-tested interview schedule consisting of questions regarding demographic background information of the study participants; assessing knowledge related to malaria etiology, mode of transmission, prevention, national drug policy; attitude relating to prioritization of malaria services; practices related to diagnosis and treatment of malaria; workload and incentives; gap between demand and supply of malaria diagnostics and anti-malarials administered. Prior to the commencement of the survey, the interview schedule (Annexure 1) was tested and standardized [4]. The interviewers had a minimum college level (graduation) of education and about 3–5 years’ work experience towards conducting community health survey. Before conducting the survey, all interviewers were provided with 1 week of training to conduct the interviews using the pre-tested standardized interview schedule. The interview was conducted in local language (Hindi). Knowledge and practice gaps were identified by analysis of data collected from the interviews of ASHAs in these three districts.

### Sample size and sampling strategy

The sample size was estimated by statistical power calculation for the binary proportion assumed that a statistical power of 0.80 and the Type 1 error rate was controlled at α = 0.05. Power calculation was generated using following formula of simple random sampling for infinite population based on 13% rates of the incorrect responses to the questions related to the knowledge and diagnosis and treatment practices on malaria as reported by Hussain et al. [9] with 5% absolute precision which determined 175 minimum number of participants was required.

$$n=\frac{{Z}^{2}p(1-p)}{{l}^{2}}$$

Further, this number was inflated to 15% non-response, which guided the survey to be conducted on 200 study participants from each study area. Further, the study participants were recruited using simple random sampling method from the different blocks of each district by using probability proportional to size technique.

### Ethical clearance

This study has been cleared by the Institutional Ethics Committee (IEC) of ICMR—National Institute of Research in Tribal Health (NIRTH) on 16 March, 2017 (reference no. 201701/10). Written informed consents were obtained from all the study participants.

### Data management and analysis

All the interview schedules were checked by the interviewers for their completeness after completing the interview in field. Further, 10% randomly selected interview schedules were validated by supervisors for illogical entries, and following the skipping patterns of questions. Numerically coded data were entered in CS-Pro 7.0 data entry application (Census and Survey Processing System, US Census Bureau and ICF International, Serpro SA) and logical expressions and conditional statements were used to minimize errors in data entry. Data analysis was done with R 4.1.2 for Windows (R Foundation for Statistical Computing, Vienna, Austria). A comparison of findings between Mandla (baseline), Balaghat and Dindori was performed in reference to Mandla (endline) using simple descriptive statistics, comparison of means and multivariate logistic regression model.

## Results

The total sample size was 220 (baseline) and 261 (endline) from Mandla, 230 from Balaghat, and 184 from Dindori. In Dindori, the number of participants was less than the designated number of 200, however it had sufficient power of analysis (beta = 0.96).

### Socio-demographic characteristics

The mean age of ASHAs from Mandla increased from 33.14 ± 5.3 in 2017 to 35.24 ± 5.92 years in 2021, due to the difference in time between the two surveys (p < 0.01). When compared to Mandla endline data, the mean age of ASHAs from Balaghat was significantly higher (p < 0.001). ASHAs of Balaghat and Mandla district had higher weightage of Scheduled Castes (14.35%) and Scheduled Tribes (74.71%), respectively, which is a socially and economically deprived class in India [10]. Amongst the three districts, Balaghat had the highest proportion of ASHAs who had achieved higher education (19.57%). The overall literacy percentage among ASHAs did not differ significantly, whereas, husbands of ASHAs among Mandla (endline) were comparatively less literate. The major source of family income was agriculture and casual labour (Table 1).

**Table 1:** Socio-demographic background characteristics of ASHA participants

Variables	Mandla		Balaghat (N = 230)	Dindori (N = 184)
	Baseline (N = 220)	End line^§^ (N = 261)		
Age in years (mean ± sd)	33.14 ± 5.3**	35.24 ± 5.92	39.18 ± 6.35***	36.21 ± 6.05
Married	220 (100.00)	261 (100.00)	230 (100.00)	184 (100.00)
No. of children (mean ± sd)	2.26 ± 1.07***	1.61 ± 1.34	2.15 ± 0.95***	2.44 ± 0.91***
0	22 (10.00)***	92 (35.25)	16 (6.96)***	7 (3.80)***
1	12 (5.45)	12 (4.60)	20 (8.70)	8 (4.35)
2	94 (42.73)**	78 (29.89)	125 (54.35)***	90 (48.91)***
> 2	92 (41.82)**	79 (30.27)	69 (30.00)	79 (42.93)**
Caste				
SC	13 (5.91)	13 (4.98)	33 (14.35)**	18 (9.78)*
ST	149 (67.73)	195 (74.71)	60 (26.09)***	121 (65.76)*
OBC	54 (24.55)	50 (19.16)	132 (57.39)***	43 (23.37)
GEN	4 (1.82)	3 (1.15)	5 (2.17)	2 (1.09)
Education				
Illiterate	3 (1.36)	3 (1.15)	0 (0.00)	0 (0.00)
Primary	21 (9.55)**	47 (18.01)	2 (0.87)***	3 (1.63)***
Middle	117 (53.18)	118 (45.21)	36 (15.65)***	86 (46.74)
Hr Sec	52 (23.64)	46 (17.62)	62 (26.96)*	60 (32.61)**
Graduate	23 (10.45)	29 (11.11)	85 (36.96)***	27 (14.67)
Higher education	4 (1.82)**	18 (6.90)	45 (19.57)***	8 (4.35)
Husband’s education				
Illiterate	21 (9.55)***	92 (35.25)	2 (0.87)***	0 (0.00)***
Primary	27 (12.27)	44 (16.86)	3 (1.30)***	5 (2.72)***
Middle	66 (30.00)**	46 (17.62)	34 (14.78)	77 (41.85)***
Hr Sec	44 (20.00)**	23 (8.81)	63 (27.39)***	61 (33.15)***
Graduate	41 (18.64)*	29 (11.11)	86 (37.39)***	30 (16.30)
Higher education	21 (9.55)	27 (10.34)	42 (18.26)*	11 (5.98)
Other source of income				
House wife	87 (39.55)***	157 (60.15)	218 (94.78)***	154 (83.70)***
Agriculture labour	51 (23.18)	60 (22.99)	10 (4.35)***	26 (14.13)*
Casual labour	27 (12.27)***	7 (2.68)	2 (0.87)	3 (1.63)
Agriculture in own land	51 (23.18)*	37 (14.18)	0 (0.00)***	0 (0.00)***
Small trade	4 (1.82)*	0 (0.00)	0 (0.00)	1 (0.54)
Forest produce collection	0 (0.00)	0 (0.00)	0 (0.00)	0 (0.00)
Husband’s occupation				
Agriculture labour	78 (35.45)	81 (31.03)	151 (65.65)***	128 (69.57)***
Casual labour	59 (26.82)***	140 (53.64)	41 (17.83)***	46 (25.00)***
Agriculture in own land	63 (28.64)***	34 (13.03)	6 (2.61)***	3 (1.63)***
Small trade	8 (3.64)*	2 (0.77)	15 (6.52)**	2 (1.09)
Forest produce collection	0 (0.00)	0 (0.00)	0 (0.00)	1 (0.54)
Other	12 (5.45)*	4 (1.53)	17 (7.39)**	4 (2.17)

^§^Reference category; *p < 0.05; **p < 0.01; ***p < 0.001

### Knowledge, attitude and practices (KAP) related to malaria

Knowledge, attitude and practices (KAP) assessment related to malaria was conducted for all ASHAs in the study. Awareness regarding the cause of malaria infection was high in all three districts, with more than 97% ASHAs reporting mosquito bites followed by a very small fraction of ASHAs reporting other causes, such as micro-organisms, contact and other insects. It was noted that the misconception of spread of malaria by consumption of contaminated water or food reduced to 7.66% in 2021 from 30.91% in 2017 in Mandla district (p < 0.001) and was even lower in Balaghat (0.43%) and Dindori (3.26%) districts, which was statistically significant (p < 0.05). More than two-thirds of ASHAs had an understanding that mosquitoes breed in water. Furthermore, knowledge had significantly improved among endline study participants of Mandla district, particularly related to breeding of mosquitoes in fresh running water (p < 0.001). The correct knowledge of high-risk groups for malaria was mostly similar in ASHAs of all three districts. However, misinformation in age group  > 60 years was found significantly higher in Balaghat (30%) and Dindori (38.04) (p < 0.001).

Information regarding children aged 2–10 years being a high-risk group for malaria was also found to be higher in all three surveys in endline Mandla (25.29%), Balaghat (22.17%) and Dindori (19.02%) compared to baseline Mandla (5.91%). Knowledge regarding preventive measures against malaria using ITN/LLIN rose from 23.64% in Mandla baseline to 37.55% in Mandla endline (p < 0.01) but was poor in Balaghat (10.87%) and Dindori (9.24%) (p < 0.001), when compared with endline Mandla data. All other unproven preventive measures viz*.* clearing stagnant water, use of smoke, coverage of body with clothes, and prophylactic medication remained lower in Mandla endline as compared to Mandla baseline (p < 0.001). All surveys showed fever was the most common symptoms of malaria. Higher coverage of population by mosquito nets was seen in Mandla endline (29.55% in 2017 to 62.45% in 2021), Dindori (72.83%) and Balaghat districts (42.61%) (p < 0.001) (Table 2).

**Table 2:** Knowledge regarding malaria etiology, transmission and preventive measures and symptoms

Variables	Mandla		Balaghat	Dindori
	Baseline	End line^§^		
	(N = 220)	(N = 261)	(N = 230)	(N = 184)
	n (%)	n (%)	n (%)	n (%)
Source of malaria infection				
Mosquito bites	215 (97.73)	259 (99.23)	229 (99.57)	180 (97.83)
Microorganism	20 (9.09)**	6 (2.30)	0 (0.00)*	3 (1.63)
Contact	13 (5.91)**	4 (1.53)	0 (0.00)	1 (0.54)
Other insects	8 (3.64)*	2 (0.77)	0 (0.00)	1 (0.54)
Consuming contaminated water/food	68 (30.91)***	20 (7.66)	1 (0.43)**	6 (3.26)*
Mosquito breeding places				
Stagnant water	184 (83.64)	202 (77.39)	185 (80.43)	149 (80.98)
Fresh or running water	28 (12.73)***	145 (55.56)	36 (15.65)***	32 (17.39)***
Garbage	7 (3.18)**	0 (0.00)	21 (9.13)***	10 (5.43)**
Dark and humid place	1 (0.45)	0 (0.00)	18 (7.83)***	25 (13.59)***
High risk group of malaria				
Infants and pregnant woman	112 (50.91)	117 (44.83)	110 (47.83)	79 (42.93)
Old age group	7 (3.18)	11 (4.21)	69 (30.00)***	70 (38.04)***
Children of 2–10 year	13 (5.91)***	66 (25.29)	51 (22.17)	35 (19.02)
All of the above	57 (25.91)	67 (25.67)	0 (0.00)***	0 (0.00)***
Preventive measures of malaria				
Plain Mosquito nets	203 (92.27)	246 (94.25)	214 (93.04)	170 (92.39)
ITN/LLIN	52 (23.64)**	98 (37.55)	25 (10.87)***	17 (9.24)***
Clear stagnant water	131 (59.55)***	91 (34.87)	62 (26.96)	30 (16.30)***
Insecticide spray	68 (30.91)**	43 (16.48)	38 (16.52)	5 (2.72)***
Repellents (coils, etc.)	55 (25.00)*	42 (16.09)	40 (17.39)	22 (11.96)
Smoke	127 (57.73)***	101 (38.70)	71 (30.87)	60 (32.61)
Skin lotions or ointments	24 (10.91)	16 (6.13)	17 (7.39)	13 (7.07)
Wear clothes to cover the body	43 (19.55)***	11 (4.21)	15 (6.52)	6 (3.26)
Take medicines for malaria	31 (14.09)***	12 (4.60)	25 (10.87)**	22 (11.96)**
Keep surroundings clean	116 (52.73)	116 (44.44)	123 (53.48)*	75 (40.76)
Common symptoms of malaria				
High fever	205 (93.18)	247 (94.64)	182 (79.13)***	132 (71.74)***
Ordinary fever	69 (31.36)***	38 (14.56)	65 (28.26)**	79 (42.93)***
Chills	162 (73.64)*	164 (62.84)	177 (76.96)**	130 (70.65)
Headache	162 (73.64)	187 (71.65)	147 (63.91)	65 (35.33)***
Bodyache	154 (70.00)***	106 (40.61)	42 (18.26)***	25 (13.59)***
Nausea/vomiting	120 (54.55)***	65 (24.90)	58 (25.22)***	27 (14.67)**
Common malaria species in area				
*P. falciparum*	99 (45.00)	116 (44.44)	120 (52.17)	95 (51.63)
*P. vivax*	60 (27.27)	65 (24.90)	81 (35.22)*	63 (34.24)*
Both (Pf and Pv)	61 (27.73)	80 (30.65)	29 (12.61)***	26 (14.13)**
Mosquito nets distributed in area	65 (29.55)***	163 (62.45)	98 (42.61)***	134 (72.83)***
Program nets were ITN/LLIN	62 (28.18)***	125 (47.89)	87 (37.83)*	79 (42.93)

^§^ Reference category; *p < 0.05; **p < 0.01; ***p < 0.001

As per the guidelines of the National Centre for Vector Borne Diseases Control (NCVBDC), formerly known as the National Vector Borne Disease Control Programme (NVBDCP); chloroquine (CQ), primaquine (PQ) and ACT are the recommended drugs for treatment of malaria. Regarding use of CQ for treatment of *P. vivax* cases, ASHAs of Dindori district lagged with 74.46% compliance (p < 0.01). Regarding use of ACT blister packs for treatment of *P. falciparum*, poorest compliance was reported by ASHAs in Balaghat district (30%, p < 0.001). A significant rise in administration of PQ for complete 14 days for radical treatment of *P. vivax* was seen from 40 to 63.22% in Mandla (p < 0.001); however, this remained significantly lower in districts Balaghat (32.61%) and Dindori (26.09%) than Mandla endline (p < 0.001). Another significant rise in 1-day administration of PQ for radical treatment of *P. falciparum* cases was seen in Mandla from 33.18 to 50.96% (p < 0.001) and also with significant differences in Balaghat (33.48%) and Dindori (24.46%) when compared to Mandla endline survey (p < 0.001).

ACT, which comes in different colour blister packs according to age group, is given for the treatment of uncomplicated falciparum malaria. ASHAs were asked to identify these coloured packs as recommended for specific age groups. Only one ASHA each from Balaghat (0.43%) and Dindori (0.54%) (p < 0.001) could correctly identify all five colour-coded ACT blister packs and the majority could not identify a single ACT blister pack in Balaghat (79.13%) and Dindori (92.39%). In Mandla, the correct identification of all five blister packs rose from 6.36% in 2017 to 56.70% in 2021 (p < 0.001) (Table 3).

**Table 3:** Knowledge regarding treatment guidelines of malaria positive cases

Variables	Mandla		Balaghat (N = 230)	Dindori (N = 184)
	Baseline (N = 220)	End line^§^ (N = 261)		
Treatment of malaria cases using anti-malarials				
Choloroquine for treatment of *P. vivax*	187 (85.00)	221 (84.67)	190 (82.61)	137 (74.46)**
ACT blister pack for treatment of *P. falciparum*	98 (44.55)	127 (48.66)	69 (30.00)***	81 (44.02)
Refer	22 (10.00)*	12 (4.60)	2 (0.87)**	0 (0.00)**
Do not treat/refer	16 (7.27)**	3 (1.15)	3 (1.30)**	9 (4.89)*
Provide primaquine				
For *P. vivax* cases	134 (60.91)**	191 (73.18)	122 (53.04)***	74 (40.22)***
For 14 days	88 (40.00)***	165 (63.22)	75 (32.61)***	48 (26.09)***
For *P. falciparum* cases	109 (49.55)**	161 (61.69)	112 (48.70)**	64 (34.78)***
For state one day	73 (33.18)***	133 (50.96)	77 (33.48)***	45 (24.46)***
Adhere to malaria treatment national policy	189 (85.91)*	240 (91.95)	194 (84.35)**	146 (79.35)***
Proportion of correctly matched the ACT colored blister pack according to the age group (Correctly matched/Total blister pack)				
0 (0/5)	145 (65.91)***	7 (2.68)	182 (79.13)***	170 (92.39)***
20 (1/5)	20 (9.09)	21 (8.05)	13 (5.65)	5 (2.72)*
40 (2/5)	27 (12.27)	36(13.79)	34 (14.78)	7 (3.80)**
60 (3/5)	12 (5.45)	17 (6.51)	0***	1 (0.54)**
80 (4/5)	2 (0.91)***	32 (12.26)	0***	0***
100 (5/5)	14 (6.36)***	148 (56.70)	1 (0.43)***	1 (0.54)***
Proportion of correctly interpreted RDT result (Correctly interpreted/Total test)				
0 (0/6)	15 (6.82)**	3 (1.15)	4 (1.74)	9 (4.89)*
16.67 (1/6)	19 (8.64)**	7 (2.68)	15 (6.52)*	22 (11.96)***
33.33 (2/6)	10 (4.55)*	4 (1.53)	8 (3.48)	5 (2.72)
50.00 (3/6)	33 (15.00)**	15 (5.75)	29 (12.61)**	12 (6.52)
66.67 (4/6)	49 (22.27)	46 (17.62)	61 (26.52)*	41 (22.28)
83.33 (5/6)	31 (14.09)	26 (9.96)	34 (14.78)	63 (34.24)***
100.00 (6/6)	63 (28.64)***	160 (61.30)	79 (34.35)***	32 (17.39)***

^§^ Reference category; *p < 0.05; **p < 0.01; ***p < 0.001

ASHAs were asked to interpret various test results of the bivalent *P. falciparum /P. vivax* malaria RDT; only 17.39% (p < 0.01) and 34.35% ASHAs from Dindori and Balaghat, respectively, could identify all six scenarios of RDTs correctly. In Mandla, the correct identification of all scenarios rose from 28.64% in 2017 to 61.30% in 2021. This improvement was statistically significant and was also higher than Balaghat and Dindori (p < 0.001) (Table 3).

### Healthcare services delivery

It was found that on an average, ASHAs in Mandla and Dindori have been working for the past 10.28 ± 4.28 and 10.99 ± 3.53 years, respectively. Whereas, in Balaghat, the average duration was reported 8.37 ± 4.72 years. Almost all ASHAs from all the districts reported to have received training in malaria diagnosis and treatment. Different methods to motivate a community to seek healthcare were used by ASHAs: door-to-door visits was reported by 100% ASHAs in Balaghat and Dindori and almost all ASHAs of Mandla district (97%); interpersonal communication was used least by ASHAs of Balaghat (8.26%) and Dindori (11.41%), and most by ASHAs of Mandla (30.65%). Government campaigns were reported by only 3.8% and 10% ASHAs of Dindori and Balaghat, respectively. A reduction in these campaigns was noted in Mandla district from 33.18% in 2017 to 10.73% in 2021 (p < 0.001). All ASHAs of Mandla endline reported having a printed guide for malaria diagnosis and treatment, whereas 69.57% and 68.70% ASHAs of Dindori and Balaghat reported having such guides (p < 0.001). In 2017, ASHAs of Mandla reported malaria and vector-borne diseases as their fifth priority (23.64%) amongst all activities assigned to them. In 2021, this priority further slipped to sixth place (9.58%, p < 0.001), and was reported as similar priority by ASHAs of Balaghat (4.78%, p < 0.05) and Dindori (3.26%, p < 0.01). Priorities of remaining health programmes, such as maternal and child healthcare and feeding, family planning, immunization, vector-borne disease including malaria, tuberculosis, and other communicable diseases remained mostly the same for all districts, with maternal and child health services as top priority for ASHAs. Diagnosis of malaria by RDTs was reported by almost all ASHAs of the three districts. However, there was a reduction in diagnosis using blood smears by ASHAs of Mandla from 83.18% in 2017 to 36.78% in 2021 (p < 0.001). This method of diagnosis was still being used by 33.91% ASHAs in Balaghat and 58.15% ASHA in Dindori districts (p < 0.001) (Table 4).

**Table 4:** Practice of health care services delivery to the community by ASHAs

Variables	Mandla		Balaghat (N = 230)	Dindori (N = 184)
	Baseline (N = 220)	End line^§^ (N = 261)		
No. of years working as ASHA	8.14 ± 3.40***	10.28 ± 4.28	8.37 ± 4.72***	10.99 ± 3.53
Received training for anyone health program	211 (95.91)**	260 (99.62)	230 (100.00)***	183 (99.46)
Received training in malaria diagnosis and treatment	196 (89.09)***	260 (99.62)	217 (94.35)**	180 (97.83)
People sought health care from you	210 (95.45)**	259 (99.23)	226 (98.26)	182 (98.91)
No. of patients visited you during last week	2.95 ± 4.24	3.09 ± 3.17	4.82 ± 4.71***	3.69 ± 3.19*
No. of patients visited you during last month	7.21 ± 8.54**	9.19 ± 7.81	13.02 ± 10.00***	9.35 ± 6.45
Promote institutional delivery	218 (99.09)	261 (100)	230(100)	184 (100.00)
No. of pregnant women take for institutional delivery during last week	0.53 ± 1.06***	0.21 ± 0.59	0.53 ± 0.85***	0.33 ± 0.64*
No. of pregnant women take for institutional delivery during last month	1.27 ± 1.61**	0.77 ± 1.65	1.56 ± 1.95***	0.92 ± 1.32
Motivate community to seek health care	218 (99.09)	261 (100)	230 (100.00)	184 (100.00)
By door to door visit	208 (94.55)	253 (96.93)	230 (100.00)**	184 (100.00)*
Inter person communication	139 (63.18)***	80 (30.65)	19 (8.26)***	21 (11.41)***
Govt. campaign program	73 (33.18)***	28 (10.73)	23 (10.00)	7 (3.80)**
Have guide for malaria diagnosis and treatment	122 (55.45)***	261 (100)	158 (68.70)***	128 (69.57)***
Top Priority of work given to				
Maternal health care	194 (88.18)***	192 (73.56)	183 (79.57)	135 (73.37)
Child health care and feeding	149 (67.73)*	150 (57.47)	133 (57.83)	122 (66.30)
Family planning	70 (31.82)	63 (24.14)	70 (30.43)	39 (21.20)
Immunization	87 (39.55)	123 (47.13)	64 (27.83)***	68 (36.96)*
Vector borne disease including malaria	52 (23.64)***	25 (9.58)	11 (4.78)*	6 (3.26)**
TB and other communicable disease	55 (25.00)	60 (22.99)	11 (4.78)***	4 (2.17)***
Malaria diagnosis provided by				
RDT	213 (96.82)*	260 (99.62)	223 (96.96)*	179 (97.28)*
Blood smear	183 (83.18)***	96 (36.78)	78 (33.91)	107 (58.15)***
Symptomatic	17 (7.73)***	0 (0.00)	0 (0.00)	4 (2.17)*
Timely receive incentives for health service promotion	99 (45.00)***	248 (95.01)	217 (94.35)	182 (98.91)*
Amount of incentive received during last 1 year	28246 ± 11,932	28654 ± 12,403	28885 ± 13,148	28212 ± 12,279
Amount of incentive received during last 3 month	1582 ± 791	1627 ± 831	1509 ± 828	1654 ± 804

^§^Reference category; *p < 0.05; **p < 0.01; ***p < 0.001

### Fever and malaria cases attended by ASHAs

Fever surveillance is the primary strategy to track, test and treat malaria cases in the community. ASHAs of Balaghat and Dindori took the lead in recordkeeping of all fever cases tested with 97.39% and 95.65%, respectively, whereas ASHAs of Mandla lagged with only 86.97% in 2021. The number of fever cases diagnosed by ASHAs of Mandla in the previous 3 months rose from 12.35 ± 16.29 to 18.68 ± 15.55 (p < 0.001) and was 23.01 ± 17.23 and 18.0 ± 10.8 by ASHAs of Balaghat and Dindori, respectively. It was intriguing to note that despite reporting zero malaria cases treated by ASHAs of Dindori district in the previous 3 months, the fever caseload was similar to Mandla district (Table 5).

**Table 5:** Record keeping practices for documentation of cases diagnosed and treated by ASHAs

Variables	Mandla		Balaghat (N = 230)	Dindori (N = 184)
	Baseline (N = 220)	End line^§^ (N = 261)		
Maintain record of fever cases diagnosed and treated	190 (86.36)	227 (86.97)	224 (97.39)***	176 (95.65)**
Number of fever cases diagnosed				
During last week	2.61 ± 4.03	2.71 ± 2.80	3.41 ± 4.03*	2.90 ± 2.68
During last month	5.96 ± 6.72**	7.72 ± 6.15	9.50 ± 8.74**	7.31 ± 5.00
During last 3 month	12.35 ± 16.29***	18.68 ± 15.55	23.01 ± 17.23**	18.0 ± 10.80
Number of *P. vivax* cases diagnosed				
During last week	0.01 ± 0.08	0	0 ± 0.07	0
During last month	0.01 ± 0.08	0 ± 0.07	0	0
During last 3 month	0.04 ± 0.33	0.01 ± 0.13	0 ± 0.07	0
Number of *P. falciparum* cases diagnosed				
During last week	0	0	0.12 ± 1.39	0
During last month	0.06 ± 0.71	0	0.08 ± 0.55*	0
During last 3 month	0.05 ± 0.49	0.02 ± 0.26	0.14 ± 0.96*	0
Number of *P. vivax* cases treated				
During last week	0.63 ± 7.85	0	0 ± 0.07	0
During last month	0.63 ± 7.85	0 ± 0.07	0	0
During last 3 month	0.64 ± 7.85	0.01 ± 0.13	0	0
Number of *P. falciparum* cases treated				
During last week	0.62 ± 7.85	0	0.02 ± 0.24	0
During last month	0.62 ± 7.85	0	0.04 ± 0.30*	0
During last 3 month	0.67 ± 7.86	0.02 ± 0.26	0.13 ± 0.95	0

^§^ Reference category; *p < 0.05; **p < 0.01; ***p < 0.001

### Logistic regression

The multivariate logistic regression with controlled effects of all exposure factors considered in the model revealed that with reference to the Mandla endline, the Mandla baseline was 0.39, 0.48, 0.34, and 0.07 times significantly lower odds for malaria-related knowledge on disease etiology, prevention, diagnosis, and treatment, respectively (p < 0.001). This suggests that during the period of MEDP, the overall malaria-related knowledge has improved significantly by 52–93% for different parameters taken into consideration. Regarding the practices of diagnosis, treatment and other health services delivery, odds were 0.25, 0.95 and 0.32%, respectively in Mandla baseline with reference to Mandla endline, but were not statistically significant. In Balaghat, ASHAs had less knowledge of malaria etiology (aOR = 0.26), prevention (aOR = 0.47), diagnosis (aOR = 0.37), and treatment (aOR = 0.01) (p < 0.001) compared with district Mandla endline. The practice of malaria diagnosis, treatment and other health service delivery was also less but not statistically significant. Similarly, in Dindori, less was noted of knowledge on malaria etiology (aOR = 0.37), prevention (aOR = 0.42), diagnosis (aOR = 0.16), treatment (aOR = 0.01) (p < 0.001), and treatment practices (aOR = 0.37, p < 0.01) compared with district Mandla endline.

Education to middle school and higher had a significant positive effect on knowledge about scientifically proven preventive measures from malaria (aOR = 1.89, p < 0.05) and an extremely significant effect on treatment practices (aOR = 32.45, p < 0.001). Belonging to Scheduled Tribes did not have any significant association with malaria knowledge or practices of ASHAs. Malaria training had a positive effect with knowledge about malaria etiology (aOR = 1.65), prevention (aOR = 1.54) and diagnosis (aOR = 1.87), however, it was not found significant statistically (p > 0.05); while practices towards malaria diagnosis (aOR = 3.29, p < 0.05), treatment (aOR = 4.74, p < 0.001), and practicing of other health programmes (aOR = 3.52, p < 0.05) had strong positive impact on received malaria training.

ASHAs having a printed guide on malaria services had a positive impact on knowledge and practices related to malaria of which knowledge of malaria diagnosis (aOR = 1.90) and treatment practices (aOR = 2.47) were highly significant (p < 0.01). Further, the length of work experience (more than 10 years) had a significant positive association with treatment practices (aOR = 1.96, p < 0.01) (Table 6).

**Table 6:** Multivariate logistic regression analysis of factors associated with knowledge about malaria and practices of ASHAs

Factors	Knowledge aOR(95% CI)				Practices aOR(95% CI)		
	Malaria etiology	Prevention	Diagnosis	Treatment	Diagnosis	Treatment	Other health service
Districts							
Mandla end line (N = 261)	Reference	Reference	Reference	Reference	Reference	Reference	Reference
Mandla baseline (N = 220)	0.39 (0.24–0.64)***	0.48 (0.32–0.72)***	0.34 (0.22–0.51)***	0.07 (0.04–0.15)***	0.25 (0.03–2.32)	0.95 (0.43–2.07)	0.32 (0.06–1.73)
Balaghat (N = 230)	0.26 (0.16–0.42)***	0.47 (0.31–0.71)***	0.37 (0.24–0.57)***	0.01 (0.001–0.04)***	0.26 (0.03–2.45)	0.60 (0.28–1.29)	0.85 (0.13–5.45)
Dindori (N = 184)	0.37 (0.23–0.59)***	0.42 (0.28–0.63)***	0.16 (0.10–0.25)***	0.01 (0.001–0.04)***	0.24 (0.03–2.23)	0.37 (0.18–0.77)**	1.07 (0.14–8.41)
Education							
Upto primary (N = 67)	Reference	Reference	Reference	Reference	Reference	Reference	Reference
Middle & higher (N = 828)	1.59 (0.93–2.71)	1.89 (1.09–3.30)*	1.52 (0.84–2.76)	0.95 (0.40–2.25)	2.46 (0.76–8.01)	32.45 (16.90–62.39)***	1.95 (0.51–7.44)
Caste							
Non ST (N = 525)	Reference	Reference	Reference	Reference	Reference	Reference	Reference
ST (N = 370)	0.97 (0.70–1.35)	1.01 (0.75–1.36)	0.84 (0.61–1.16)	1.61 (0.97–2.67)	1.66 (0.62–4.44)	0.71 (0.43–1.19)	1.27 (0.45–3.56)
Received training in malaria							
No (N = 32)	Reference	Reference	Reference	Reference	Reference	Reference	Reference
Yes (N = 863)	1.65 (0.91–3.01)	1.54 (0.80–2.96)	1.87 (0.84–4.16)	0.93 (0.17–4.90)	3.29 (1.06–10.21)*	4.74 (2.32–9.67)***	3.52 (1.07–11.52)*
Having malaria learner’s guide							
No (N = 205)	Reference	Reference	Reference	Reference	Reference	Reference	Reference
Yes (N = 690)	1.22 (0.86–1.72)	1.05 (0.74–1.48)	1.90 (1.27–2.84)**	2.49 (0.76–8.09)	2.15 (0.81–5.68)	2.47 (1.47–4.13)**	1.61 (0.56–4.67)
Length of experience							
< 10 years (N = 445)	Reference	Reference	Reference	Reference	Reference	Reference	Reference
> = 10 years (N = 450)	1.10 (0.81–1.51)	1.20 (0.90–1.60)	0.95 (0.70–1.30)	1.16 (0.70–1.90)	1.49 (0.59–3.81)	1.96 (1.23–3.13)**	2.54 (0.91–7.11)

*aOR* Adjusted Odds Ratio, *CI* Confidence Interval; *p < 0.05; **p < 0.01; ***p < 0.001

## Discussion

This study was performed to determine the needs-assessment of malaria diagnosis and treatment for ASHA workers of Mandla after 3 years of MEDP activities in the district and to compare baseline [4] and endline findings for Mandla with two adjoining districts of Balaghat and Dindori. The findings of the study were grouped into socio-demographic characteristics: KAP related to malaria; healthcare services delivery; fever and malaria cases attended by ASHAs; effect of districts, education, caste, training, printed guide, and length of experience on knowledge and practices of ASHAs of the three districts using multivariate logistic regression model.

The mean age of ASHAs in Mandla increased from 33.14 years during baseline survey to 35.24 years during endline survey, which was due to the duration between the two surveys. As per the guidelines by the National Health Mission, minimum educational qualification for recruitment of an ASHA is tenth grade or high school. This educational condition gets relaxed in situations where suitable persons with prescribed qualifications are not available in a village [11]. At least 8 years of schooling were reported in Karnataka by 90%, 82.6% in Bihar, and 85.75% in Odisha [9, 12]. The highest percentage observed in Karnataka may be due to the better literacy scores of the State [13]. This criteria has been relaxed in Mandla where more than half of the ASHAs had up to middle school qualification only. It was interesting to note that Balaghat and Dindori districts had only 15% and 44% ASHAs, respectively, who were not educated up to high school.

This study revealed that higher education had a significant positive impact on treatment practices of ASHAs (aOR = 32.45), but ASHAs of Mandla associated with a significant positive impact on all domains of knowledge and practices compared to ASHAs of Dindori and Balaghat. This could be due to the periodic training provided by MEDP in Mandla, which decreased the effect of lesser education amongst ASHAs and helped them score better in all domains of malaria diagnosis of treatment [4, 5]. Similar effect of training was noted on the Village Malaria Workers of MEDP [14]. A study conducted in China reported the importance of the implementation of a sustainable malaria training programme to healthcare providers until the elimination goal has been achieved. Peripheral health workers expected to receive more frequent training in field work coordination and on specific public health action with regard to case detection and focus investigation [15]. While levels of correct knowledge regarding source of malaria infection remained extremely good amongst ASHAs of all districts, reduction in misconceptions related to spread of malaria was noted in Mandla along with increase in knowledge regarding preventive measures using ITN/LLIN. This improvement could be attributed to the robust training curriculum administered to ASHAs of Mandla district by MEDP, which was developed on the same lines of MEDP’s training curriculum for its Village Malaria Workers and Malaria Field Coordinators [14]. Another study conducted in Somalia reported a significant improvement in knowledge and practice to the national malaria guidelines in the diagnosis, treatment and prevention of malaria in the in-service training programme of frontline healthcare workers [16]

Also noted was an increase in information about high-risk age groups for malaria in all three districts. Amongst several other methods, reduction in preventive measures such as insecticide sprays was also noted in all three districts. A possible explanation for this could be the phasing out of indoor residual sprays (IRS) and introduction of LLINs to cover maximum population, resulting in overall reduction in malaria cases. The higher coverage of LLINs in Dindori district (72.83%) could be a major factor in reduction of malaria transmission in this district.

In Mandla, the drop in ranking of malaria services by ASHAs could be because of the services provided by MEDP [5, 6], thus further decreasing the malaria workload from ASHAs. However, similar rankings were noted in Balaghat and Dindori districts. While this finding from Dindori can be attributed to low malaria burden, it does not explain the reason behind poor ranking of malaria in Balaghat, which is a district with high malaria endemicity and needs to be explored further. A study done by Fathima et al. in Karnataka agreed with the results from this study where more than 80% of ASHAs reported Maternal and child health-related work as their top priority and malaria was reported by only 32% as an important activity.

The reduction in use of blood smears as a diagnostic method for malaria was noted in Mandla endline survey. This finding is aligned with the recommendation of the NCVBDC, where it is stated that either RDT or blood smears can be used for diagnosis and treatment of malaria in a community setting. With the high reliability and less turn-around-time of RDTs, MEDP advocated this method in Mandla district and was able to achieve significant improvement [5, 6]. The study also noted a reduction in interpersonal communication and government Information, Education, Communication (IEC) and Behavior Change Communication (BCC) campaigns to promote health-seeking behaviour in the community. In Mandla, this reduction could be attributed to robust active surveillance mechanism and IEC/BCC strategies employed by MEDP in the entire district [5, 17].

The improvement from 2017 to 2021 towards correct interpretation of RDTs and ACT blister packs in Mandla can be directly attributed to MEDP Mandla. However, these findings were still not at par with the performance of ASHAs in Odisha, where the ability to interpret an RDT correctly was 86.8% [9]. Given the issue of poor literacy and wrong interpretation of ACT blister packs, it would be advisable that the age group should also be mentioned in the regional language on the packet of the ACT blister packs.

The worrying results of wrong interpretation of RDTs and ACT in Balaghat and Dindori raises the question of appropriate diagnosis and treatment capabilities of ASHAs, which is very significant for malaria case reporting as well as for the continued transmission of malaria in these two districts. The study noted that despite reporting zero malaria cases in Dindori district, the incidence of fever reported by ASHAs had not decreased. Given new information about the capabilities of ASHAs in Dindori district, the claim of no malaria cases needs to be investigated. There is also a need to investigate other causes of fever in this district.

## Conclusion

This study conducted a comparative assessment of before-and-after MEDP intervention for ASHAs of Mandla, and a cross-sectional view of ASHAs of Balaghat and Dindori. The findings unequivocally established significant improvement in the overall knowledge and practices of ASHAs in Mandla district after MEDP intervention. While there is improvement in Mandla, more efforts in robust training, monitoring and supervision, on-spot inspections, accountability framework, and a sound supply chain management system are needed to further help these frontline health staff. Learning from Mandla district can be extremely helpful in improving the quality of ASHAs in Dindori, Balaghat, and other malaria-endemic districts in India. Further, the finding of zero malaria cases in Dindori are not supported by the level of knowledge and practices of ASHAs and the fever cases burden of the district. The study also points to the importance of additional indicators, such as programme management, supply chain management, inter-sectorial coordination, collection of high-quality data, and evidence-based interventions. The study informs the need to consider inadequate capacity of ASHAs to detect and treat malaria in the overall strategy for meeting 2030 malaria elimination goals.

## Data Availability

All the findings are reported in this manuscript. The hardcopy data are stored at MEDP Office, Indian Council of Medical Research-National Institute of Research in Tribal Health (ICMR-NIRTH), Jabalpur, Madhya Pradesh. Softcopy data is available on the project server of MEDP hosted by Microsoft Azure. If anyone wants to review or use the data, they should contact:

## Abbreviations

ACT: Artemisinin-based combination therapy
ASHA: Accredited Social Health Activist
BCC: Behavior change communication
CQ: Chloroquine
FDEC: Foundation for Disease Elimination and Control of India
GoMP: Government of Madhya Pradesh
ICMR: Indian Council of Medical Research
IEC: Institutional Ethical Committee
KAP: Knowledge, attitude and practices
MCH: Maternal and child health
MEDP: Malaria elimination demonstration project
NHM: National Health Mission
NIRTH: National Institute of Research in Tribal Health
NVBDCP: National Vector Borne Disease Control Programme
OBC: Other backward caste
PQ: Primaquine
RDT: Rapid diagnostic test
SC: Scheduled caste
SPSS: Statistical package for social sciences
ST: Scheduled tribe
WHO: World Health Organization
